# Validation and Adaptation of the Interpersonal Behaviors Questionnaire in the Spanish Sport Context: The Inclusion of Novelty and Analysis of Coach–Athlete Perspectives

**DOI:** 10.1002/brb3.71419

**Published:** 2026-04-16

**Authors:** José M. Aguilar‐Parra, Rubén Trigueros, Carmen Fernandez‐Ortega, Maria del Mar Miralles‐Dionis

**Affiliations:** ^1^ Department of Psychology, Hum‐878 Research Team, Health Research Centre University of Almeria Almeria Spain

**Keywords:** basic psychological needs, interpersonal behavior, motivation, physical activity

## Abstract

**Background/Purpose:**

The present study had a fourfold purpose: (a) to validate the interpersonal behaviors questionnaire (IBQ) and IBQ‐Self, (b) to compare measurement models with and without the inclusion of the novelty construct, (c) to analyze the convergence/dissonance between coach and athlete perceptions, and (d) to examine the predictive validity of the model regarding athletes’ motivation and intention to be physically active.

**Methods:**

Participants were 2085 individuals (1587 athletes and 498 coaches). Data were analyzed using descriptive statistics, reliability analysis, confirmatory factor analysis (CFA), and structural equation modeling (SEM).

**Results:**

CFA supported the structural validity of the IBQ, showing better fit indices for the hypothesized model including novelty (e.g., CFI = 0.97, TLI = 0.97, and RMSEA = 0.042). Regarding coach–athlete perceptions, rather than asymmetry, the results demonstrated a significant alignment between both perspectives. Finally, the SEM confirmed the predictive utility of the model, where satisfaction of basic psychological needs positively predicted motivation (β = 0.43 and p < 0.001), which in turn positively predicted the intention to be physically active (β = 0.39 and p < 0.001).

**Conclusion:**

The IBQ with the addition of novelty is a reliable instrument that structurally improves the original scale, demonstrating that athletes’ perceptions of the coach and coaches’ self‐perceptions are synchronized and play a key role in predicting physical activity intentions.

## Graphical abstract

This study validates the Spanish version of the Interpersonal Behaviors Questionnaire (IBQ and IBQ‐Self) by incorporating the novelty construct into the assessment of coach‐athlete dynamics. Results demonstrate significant alignment between coach self‐perceptions and athlete perceptions. The model confirms that satisfying basic psychological need—sincluding novelty—positively predicts autonomous motivation, which subsequently enhances the future intention of athletes to remain physically active.

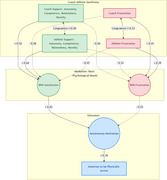

## 1 Introduction

Current research in physical activity emphasizes the critical role of the social environment in fostering adherence and sustained participation (Lindsay et al. 2017). Within this context, coaches and sports instructors are primary drivers of positive or negative experiences, directly influencing athletes’ motivation, well‐being, and active participation through their interpersonal behaviors (Yang et al. 2020). To accurately analyze these dynamics, it is essential to have validated assessment tools. One of the main theories explaining the influence of social and contextual factors on individuals is self‐determination theory (SDT; Ryan and Deci 2017).

SDT considers the influence of the social environment as an element for the innate growth and pursuit of well‐being of human beings (Ryan and Deci 2017). Thus, the social context can influence the individual, through any socializing agent, from six types of interpersonal behaviors based on the support and frustration of their psychological needs (Vansteenkiste et al. 2020). Support‐focused behaviors are primarily: autonomy, which consists of providing opportunities for free choice, encouraging free participation in various activities, and acknowledging and accepting the opinions of others; relationships, which consists of establishing a close attachment bond with others based on mutual understanding, support, and concern; and competence, which consists of successfully performing a specific task or exercise, providing useful and positive feedback, and overcoming challenges. In contrast, frustration‐focused behaviors are primarily: autonomy, which refers to coercive, malicious, and intimidating language and the imposition of demands that lack justification; competence, which refers to futile participation in activities that are complicated, questioning one's ability and inability to overcome obstacles and challenges; and relationships, which involves showing aversion and hostility towards others (Alexe et al. 2022; Bartholomew et al. 2010).

However, in recent years, different studies have emerged suggesting the incorporation of a fourth psychological need, novelty (e.g., González‐Cutre et al. 2016, 2020). According to the results of these investigations (González‐Cutre et al. 2016, 2020; Trigueros, Maldonado, et al. 2020; Trigueros et al. 2023), the need for novelty seems to be an inherent desire that exists in all cultural contexts and developmental stages and that is connected to the optimal growth of individuals, thus fulfilling some of the rules of inclusion established by Ryan and Deci (2017) in relation to the needs of new candidates. From the support and frustration disposition, the support of novelty would refer to having experiences that involve something new and that deviate from the daily routine. Frustration with novelty, on the other hand, refers to experiencing repetitive and low‐value routines all too often.

Although SDT provides the framework, it is essential to shift the focus from general principles to the specific interpersonal dynamics between coaches and athletes (Alexe et al. 2021). Current literature establishes that need‐supportive and need‐frustrating behaviors are not merely opposites but distinct interpersonal styles with specific consequences for the athlete's development (Trigueros and Garcia‐Mas 2025; Vansteenkiste et al. 2020). In the daily practice of coaching, this implies that a lack of support does not automatically equate to frustration. For instance, a coach might fail to provide sufficient feedback or autonomy (low support) without necessarily employing coercive or intimidating methods (need frustration). This distinction is crucial because it suggests that a coach's profile is not unidimensional; a sports professional might be neutral in their support yet actively frustrating in other aspects. Therefore, to capture the full reality of the sports experience; covering both the “bright side” (growth) and the “dark side” (maladaptation), it is essential to evaluate these behaviors as separate but coexisting dimensions of the coach's leadership style.

Most of the studies to assess the coach's supportive behaviors and frustration of needs have been based only on measures focused on the coached's view and not on coach‐based self‐measures (Fernández‐Batanero et al. 2019). In this sense, it is interesting to know both sides of the message that is transmitted in the form of instructions, that of the coaches and that of the athletes, as has been done with other sport variables, such as cooperation (Kılıç and Ince 2023). Thus, to assess respondents’ perceptions of autonomy, competence, and relationship supportive and frustrating behaviors, Rocchi et al. (2017) developed the interpersonal behavior questionnaire (IBQ). The instrument was created to assess how the interpersonal behaviors of a particular socializing agent are perceived, as well as how someone reports their own interpersonal behaviors (Self). Psychometric results of the scale have shown that the factor structure of the scale consists of 24 items and six factors. In addition, evidence was provided to support gender invariance of the measure, item consistency or internal reliability (with Cronbach's alpha scores between 0.75 and 0.81), and criterion validity. The IBQ has been widely extended to other contexts (Alexe et al. 2023; Tóth‐Kiraly et al. 2022; Xiao and Toyama 2020) including the Spanish context in Physical Education (Burgueño and Medina‐Casaubón 2021). However, all these versions do not explore the inclusion of the influence of the social context on the support and frustration of the psychological need for novelty. Furthermore, most of the current studies have focused on perceptions of interpersonal behavior from the viewpoint of a single social actor, either from the point of view of the student/athlete (González‐Cutre et al. 2020; Trigueros et al. 2023) or the teacher/coach (Sabiston et al. 2020; Sullivan et al. 2012). As these studies are based on a single point of view, there may be discrepancies in understanding the influence of social context on psychological needs or motivation. Thus, studies that have focused on students’ and athletes’ perceptions of teachers’, family members’ or coaches’ behaviors differ from teachers’, family members’ or coaches’ self‐perceptions of their own behaviors (Admiraal et al. 2019; Bhavsar et al. 2019; Delrue et al. 2019; Haerens et al. 2015; How et al. 2013), highlighting factors such as the association of the two views or their alignment.

Therefore, to address the identified gaps in the literature and broaden the field of study focused on interpersonal behavior in physical activity, the present study had a fourfold purpose: (a) to examine the psychometric properties and validate the IBQ and IBQ‐Self in the Spanish sport context; (b) to compare the theoretical models with and without the inclusion of novelty; (c) to explore the convergence or dissonance between coaches’ and athletes’ perceptions; and (d) to test the predictive validity of the retained model on athletes’ motivation and future intention to be physically active.

## 2 Method

### 2.1 Participants

Participants were recruited using a non‐probability convenience sampling method. It is important to note that the data comprise two independent samples; they are not matched coach–athlete dyads. The athletes’ sample consisted of 1587 participants (794 women and 793 men) with a mean age of 38.67 years (SD = 9.14). These participants were engaged in non‐elite competitive or amateur contexts, drawn from various sports (e.g., football, basketball, athletics, and volleyball) in Andalusia, Spain. The coaches’ sample comprised 498 participants (237 women and 261 men) from Andalusia, Spain, with a mean age of 34.10 years (SD = 4.35). The inclusion criteria were (a) practicing physical activity at least 3 days per week, (b) a practice duration of at least 6 months, and (c) providing informed consent. Exclusion criteria included being a high‐performance or professional athlete (to avoid heterogeneity in motivational profiles regarding recreational practice) and having any injury or condition preventing regular practice at the time of data collection.

On the other hand, the study included 498 coaches (237 women and 261 men) from Andalusia (Spain). The mean age was 34.10 years (SD = 4.35). Regarding their expertise, although a minimum of 1 year of experience was established as an inclusion criterion to ensure familiarity with at least one full annual training cycle, the selected sample possessed significantly higher experience. Specifically, the coaches reported an average professional experience of 3.45 years (SD = 1.20). Therefore, the inclusion criteria were being currently active, having more than 1 year of experience, and providing informed consent.

### 2.2 Measurements

#### 2.2.1 Coach Interpersonal Behaviors

In order to analyze the athletes’ perception of the interpersonal behavior of their coach, the IBQ of Rocchi et al. (2017). The scale consists of 24 items divided into six factors: autonomy support, competence support, relationship support, autonomy frustration, competence frustration, and relationship frustration. In addition, eight of the 16 items described in the procedure section were added, four for novelty support and four for novelty frustration. Participants completed the scale of agreement with each statement using a 7‐point scale ranging from 1 (do not agree at all) to 7 (strongly agree).

#### 2.2.2 Interpersonal Coach Self‐Behaviors

To analyze coaches’ perception of their own interpersonal behavior, the IBQ‐Self by Rocchi et al. (2017) was used. The scale consists of 24 items divided into six factors: autonomy support, competence support, relationship support, autonomy frustration, competence frustration, and relationship frustration. In addition, eight of the 16 items described in the procedure section were added, four for novelty support and four for novelty frustration. Participants completed the scale of agreement with each statement using a 7‐point scale ranging from 1 (do not agree at all) to 7 (strongly agree).

#### 2.2.3 Basic Psychological Needs

The scale of basic psychological needs towards physical activity by Trigueros, Álvarez, et al. (2020) was used. This scale is composed of two factors (satisfaction and frustration) and each factor has four sub‐factors (novelty, competence, relatedness, and autonomy). In total, the scale is made up of 35 items spread across the 8 sub‐factors. Participants in the study filled in a Likert‐type scale, with answers ranging from 1 (not true at all) to 7 (completely true).

#### 2.2.4 Motivation

The behavior regulation in practice questionnaire (BREQ‐3) by González‐Cutre et al. (2010) was used. The scale was headed by the following heading, “I do physical exercise…” The scale is made up of 6 factors (intrinsic motivation, integrated regulation, identified regulation, introjected regulation, external regulation, and demotivation) and 23 items. The questionnaire is of the Likert type, ranging from 0 (nothing true) to 4 (totally true).

For the present study, the self‐determination index (SDI) was calculated. This score is obtained from the different types of motivation that make up the BREQ‐3 scale. To calculate this index, a weight is assigned to each type of motivation according to its position on the self‐determination continuum. Thus, a weight of +3 is assigned to intrinsic regulation, +2 to integrated regulation, +1 to identified regulation, −1 to introjected regulation, −2 to external regulation, and −3 to amotivation (Vallerand 2007). The final score of the index was calculated by adding all the results obtained by multiplying the score for each of the types of motivation by their corresponding weight.

#### 2.2.5 Intention to Be Physically Active

The subscale belonging to the TPB (Hagger et al. 2009) was used. This scale consists of three items (e.g., I intend to engage in active sports and/or physical activities during my free time in the next 5 weeks…). The scale is a Likert‐type scale of up to 7 points from 1 (strongly disagree) to 7 (strongly agree).

### 2.3 Procedure

Initially, prior to the confirmatory factor analysis (CFA), it was necessary to elucidate the items referring to support and frustration of novelty for the IBQ and the IBQ‐Self through a rigorous content validity process. The method used was the Delphi method (Fink‐Hafner et al. 2019). Specifically, a panel consisting of seven experts in sports psychology and psychometrics evaluated the initial pool of items. The experts assessed each item for clarity, theoretical relevance, and representativeness regarding the novelty construct in the SDT framework. Items that achieved a minimum consensus of 80% agreement among the experts were retained. In this way, the group agreed on 16 items with the intention of developing and including a range of experiences focused on interpersonal behavior centerd on novelty relevant to the context of physical activity.

Subsequently, it was necessary to translate each of the IBQ and IBQ‐Self items into Spanish. For the translation, the procedure established by Hambleton (1994) was followed, which is based on the direct and reverse translation of the items. Thus, a group of translators with experience in the field of physical activity translated the initial items into Spanish and then another group translated the same items from Spanish into English. From this final version, the wording of the items was compared with those of the original questionnaire, checking their equivalence. Once the Spanish questionnaire was obtained and the items focused on novelty were incorporated, a group of psychologists from sport field modified those items to adapt them to the sociocultural reality of the Spanish context. This adaptation was crucial because the Spanish sporting environment differs from the original North American context, particularly regarding communication styles and interpersonal closeness. In Spain, coach–athlete interactions are often characterized by higher emotional expressiveness and more direct communication (Izquierdo and Anguera 2021; Moreno‐Murcia et al. 2019). Therefore, the wording was adjusted to ensure the items captured these nuances of warmth and directness typical of the Spanish culture, rather than a literal translation that might appear too formal or distant for the target population.

### 2.4 Data Analysis

The following statistical packages were used for the present study: SPSS v20 and Mplus v7.4. Descriptive statistics (mean, standard deviation, and Pearson correlations) were analyzed. In addition, the reliability of each of the scales was analyzed, using three parameters: McDonald's Omega Coefficient, the mean variance extracted, and Cronbach's Alpha. Moreover, the heterotrait–monotrait correlations (HTMT) of the latent factors were estimated to demonstrate their suitability in terms of specificity and sensitivity with respect to other methods, especially in large samples (Henseler et al. 2014).

Regarding the estimation method for the CFA and structural equation modeling (SEM), although WLSMV is often recommended for ordinal data, maximum likelihood (ML) estimation with robust standard errors is considered appropriate and robust for Likert scales with five or more response categories, particularly with large sample sizes (Finney and DiStefano 2013). To control for common method bias (CMB), procedural remedies were applied during data collection, such as ensuring absolute anonymity and emphasizing that there were no right or wrong answers. Additionally, Harman's single‐factor test was conducted, showing that a single factor accounted for only 18% of the total variance, which is well below the 50% threshold, indicating that CMB was not a pervasive issue in this study (Podsakoff et al. 2003). In addition, 95% bias corrected bootstrap CIs (95% CIBC) were calculated for each of the proposed pathways with 6000 bootstrap samples (Hayes and Scharkow 2013) in the CFA and SEM. For both the CFA and SEM, the following fit indices were used to accept or reject the model according to the parameters set by Hair et al. (2017): SRMR and RMSEA will show a good fit when the score is equal to or below 0.06; incremental indices (TLI, IFI, and CFI) when the score is above 0.95; *χ* ^2^/df when the score is between 2 and 3.

This study is endorsed by the Bioethics Committee of the University of Almeria (. 007/2024 UALBIO), respecting at all times the protocols established in the Declaration of Helsinki and the American Psychology Associations.

## 3 Results

### 3.1 Confirmatory Factor Analysis and Reliability Analysis

Table 1 shows the comparison between the fit indices of the IBQ scale without the addition of the novelty and the IBQ scale with the addition of the novelty. The fit indices for both scales were good, although slightly better with the addition of the novelty.

**TABLE 1:** Confirmatory factor analysis (CFA) comparisons about interpersonal behaviors questionnaire (IBQ).

Scales	*χ* ^2^	df	*χ* ^2^/df	*p*	CFI	TLI	IFI	RMSEA (90% CI)	SRMR
IBQ without novelty	696.19	237	2.94	0.001	0.95	0.95	0.95	0.051	0.039
IBQ with novelty	1257.56	436	2.89	0.001	0.96	0.96	0.96	0.044	0.034

The correlations between the IBQ factors without novelty ranged from −0.35 to 0.58, all being significant at least 0.01. Standardized regression weights were statistically significant (*p* < 0.001), ranging from 0.70 to 0.82.

The correlations between the IBQ factors with novelty ranged from −0.46 to 0.67, all being significant at least 0.01. Standardized regression weights were statistically significant (*p* < 0.001), ranging from 0.75 to 0.84.

Table 2 shows the comparison between the fit indices of the IBQ‐Self scale without the addition of the novelty and the IBQ‐Self scale with the addition of the novelty. The fit indices for both scales were good but better with the addition of the novelty.

**TABLE 2:** Confirmatory factor analysis (CFA) comparisons about interpersonal behaviors questionnaire (IBQ)‐Self.

Scales	*χ* ^2^	df	*χ* ^2^/df	*p*	CFI	TLI	IFI	RMSEA (90% CI)	SRMR
IBQ‐Self without novelty	721.02	237	3.04	0.001	0.95	0.95	0.95	0.057	0.041
IBQ‐Self with novelty	1006.51	436	2.31	0.001	0.97	0.97	0.97	0.042	0.033

***p < 0.001.

The correlations between the IBQ‐Self factors without novelty ranged from −0.46 to 0.62, all being significant at least 0.01. Standardized regression weights were statistically significant (*p* < 0.001), ranging from 0.59 to 0.80.

The correlations between the IBQ‐Self factors with novelty ranged from −0.39 to 0.60, all being significant at least 0.01. Standardized regression weights were statistically significant (*p* < 0.001), ranging from 0.74 to 0.84.

### 3.2 Descriptive Statistics

Table 3 shows the mean, standard deviation, reliability estimators (Cronbach's alpha, Omega coefficient, and HTMT), and bivariate correlations (upper diagonal). The latent correlations between the study variables were almost all significant, ranging from −0.42 to 0.71.

**TABLE 3:** Descriptive statistics, reliability analysis, bivariate correlations, and heterotrait–monotrait (HTMT).

Factors	Mean	SD	*α*	*ω*	1	2	3	4	5	6	7	8
1. IB support psychological needs (S)	5.75	1.02	0.81	0.79	—	−0.42**	0.71***	−0.37*	0.64***	−0.29**	0.24*	0.20*
2. IB frustration psychological needs (S)	1.69	0.96	0.80	0.78	−0.42	—	−0.38**	0.63***	−0.32***	0.55***	−0.18**	−0.14**
3. IB‐self support psychological needs (C)	6.12	0.77	0.84	0.81	0.67	−0.38	—	−0.25**	0.56***	−0.22**	0.31**	0.28***
4. IB‐self frustration psychological needs (C)	1.88	0.93	0.85	0.82	−0.45	0.71	−0.39	—	−0.45***	0.43**	−0.39**	−0.22***
5. Satisfaction BPN	5.22	1.17	0.81	0.80	0.59	−0.46	0.62	−0.33	—	−0.18**	0.35**	0.17*
6. Frustration BPN	1.76	1.14	0.78	0.79	−0.41	0.63	−0.53	0.65	−0.31	—	−0.20***	−0.10
7. SDI	15.33	5.22	—	—	0.52	−0.39	0.40	−0.41	0.38	−0.40	—	0.41**
8. IPA	4.61	1.72	0.80	0.78	0.30	−0.51	0.36	−0.32	0.44	−0.33	0.29	—

***p < 0.001.

Abbreviations: BPN, basic psychological needs; IB, interpersonal behavior; IPA, intention to be physically active; SDI, self‐determination index.

### 3.3 SEM: A Predictive Analysis

**FIGURE 1:**
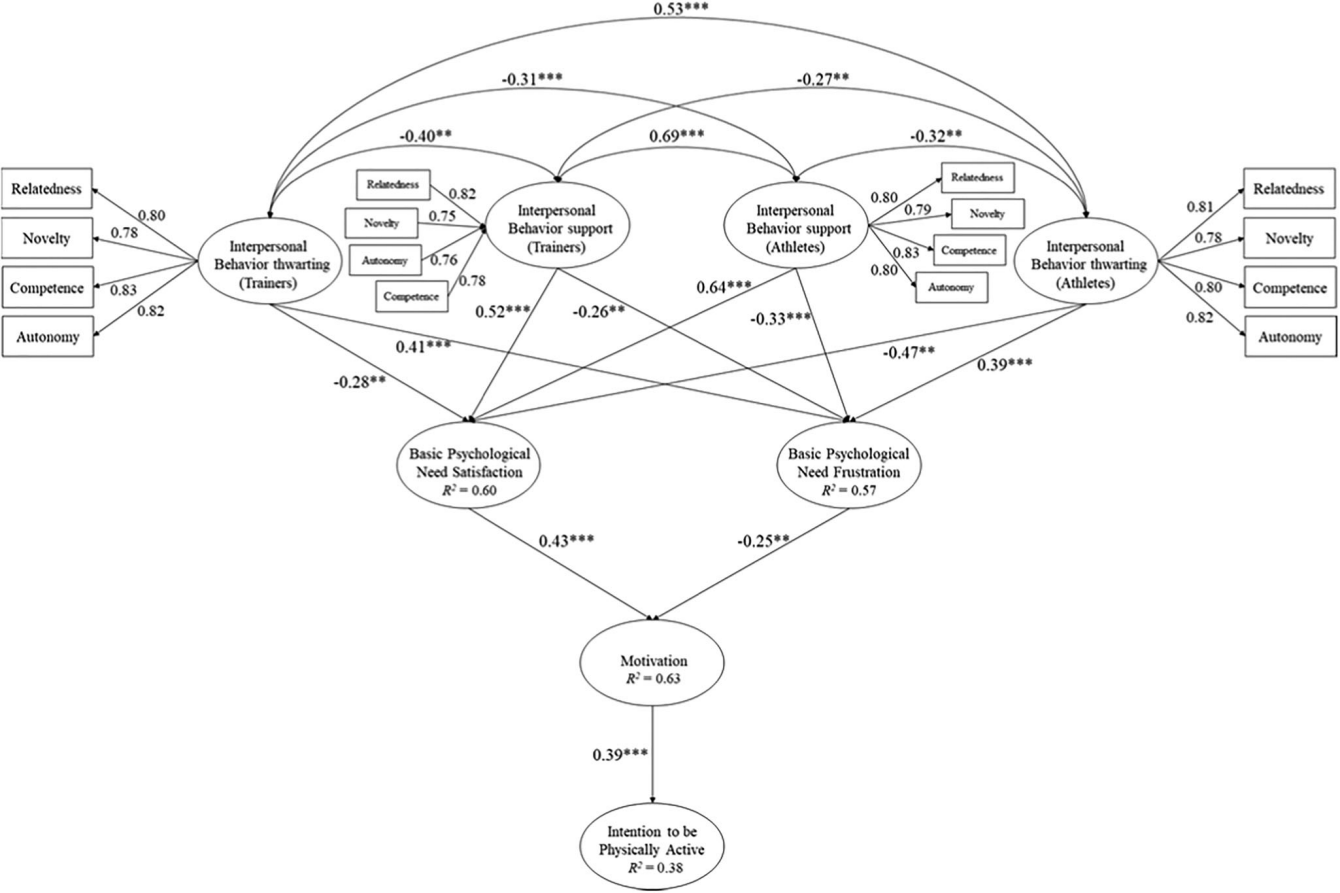
Hypothesized structural equation model.

As shown in the SEM results, the hypothesized model of the present study obtained a good fit in the different statistics (see Table 4). Furthermore, Figure 1 shows how the students’ perceived supportive interpersonal behaviors and the coach's supportive interpersonal behaviors were positively related to the satisfaction of basic psychological needs (*β* = 0.64–0.52) and negatively related to the frustration of basic psychological needs (*β* = −0.47 to −0.28). In contrast, students’ perceived interpersonal behaviors of frustration and the coach's interpersonal behaviors of frustration were positively related to the frustration of basic psychological needs (*β* = 0.39–0.41) and negatively related to the satisfaction of basic psychological needs (*β* = −0.47 to −0.28).

**TABLE 4:** Structural equation modeling (SEM) adjustment indices.

Scales	*χ* ^2^	df	*χ* ^2^/df	*p*	CFI	TLI	IFI	RMSEA (90% CI)	SRMR
Hipotetized model	1105.36	478	2.31	0.001	0.97	0.97	0.97	0.042 (0.039–0.045)	0.033
First alternative model	1129.84	360	3.14	0.001	0.95	0.95	0.95	0.057 (0.053–0.060)	0.041
Second alternative model	5688.32	1080	5.27	0.007	0.80	0.78	0.80	0.084 (0.082–0.086)	0.062

On the other hand, satisfaction of basic psychological needs was positively related to autonomous motivation (*β* = 0.43) and negatively related to frustration of basic psychological needs (*β* = −0.25). Finally, motivation was positively related to intention to be physically active (*β* = 0.39). As for the independent variables of the hypothesized model, there were variables that correlated positively with each other, being interpersonal behavioral support (coaches)‐interpersonal behavioral support (athletes) *r* = 0.69, *p* < 0.001; interpersonal behavioral frustration (coaches)‐interpersonal behavioral frustration (athletes) *r* = 0.53, *p* < 0.001. On the other hand, and complementary to the above, there were variables that correlated negatively with each other, being interpersonal behavioral support (coaches)‐interpersonal behavioral frustration (coaches) *r* = −0.40, *p* < 0.01; interpersonal behavioral support (athletes)‐interpersonal behavioral frustration (athletes) *r* = −0.32, *p* < 0.01; interpersonal behavioral support (coaches)‐interpersonal behavioral frustration (athletes) *r* = −0.27, *p* < 0.01; interpersonal behavioral support (athletes)‐interpersonal behavioral frustration (coaches) *r* = −0.31, *p* < 0.001.

Two alternative models were tested to ensure the adequacy of the proposed hypothesized model. The first alternative model (see Figure S1), which did not include novelty in supportive and frustrating interpersonal behaviors, showed a good fit to the data (see Table 2) and explained 56% of the variance in motivation, 51% of basic psychological need satisfaction, and 53% of basic psychological need frustration. However, the hypothesized model (i.e., Figure 1) showed more variance (i.e., >7%, >9%, and >4%) than the alternative model one. In addition, the hypothesized model (i.e., Figure 1) showed lower values of *χ* ^2^/df, RMSEA, SRMR, and higher values of CFI, IFI, and TLI (see, Table 4). These analyses reinforce the relevance of the proposed model, which allows us to analyze the effect of novelty on interpersonal behaviors, with all the impact this has on the development of training and the instructions that underpin it. Similarly, the second alternative model (see Figure S2), which did not include any common variables for both support and frustration of interpersonal behavior, showed a poor fit to the data (see Table 2). In this regard, the fit indices referred to the *χ* ^2^/df, SRMR, RMSEA, and incremental index values exceeded or were below the cut‐off score set by Hair et al. (2017). In this model, the variance explained was lower than in the original model.

## 4 Discussion

The present study aims to show evidence of the factor structure of the IBQ and IBQ‐Self in the context of physical activity in sport. Moreover, studies to date in the educational context have shown that sometimes the athletes’ perspective on their coaches differs from the coaches’ self‐perception of themselves, and therefore the aim was to analyze the relationship between both perspectives, coaches and athletes, analyzing their strong association, and their relationship with basic psychological needs—including novelty, motivation, and the intention to be physically active.

First, the results of both the IBQ and IBQ‐Self CFAs towards physical activity have shown that the factor structures of the questionnaires supported a 28‐item, 8‐factor correlated model that assesses each of the types of interpersonal behaviors proposed in Rocchi et al. (2017) scale plus the inclusion of novelty support and frustration in each of the scales. In addition, the SEM showed evidence in favor of a two‐factor hierarchical model that groups autonomy support, novelty, competence, and relatedness behaviors into need support behaviors and autonomy frustration, novelty competence, and relatedness behaviors into need frustration behaviors. Thus the CFA and SEM results suggest that the instrument can be used both to measure each of the eight specific types of interpersonal behaviors proposed separately and to measure the two general interpersonal behaviors (i.e., need‐supportive behaviors and need‐frustrating behaviors). In addition, the results of the CFAs showed that adjustment scores were better with the addition of novelty in both the IBQ and the IBQ‐Self. In this sense, following SDT, it is suggested that novelty is related to the pursuit of personal growth through the search for new experiences (i.e., new and different activities) and broadening (gaining more experience in what is already familiar) (Gordon and Luo 2011), which is essential for the proper development of the individual's basic psychological needs. In relation to other variables, it would be related to mastery of the environment or openness to other people as factors of psychological well‐being (Bhavsar et al. 2019) or the perception of competence as a factor of sport commitment (Camire et al. 2020).

In comparison with the validation study of the IBQ and IBQ‐Self (Rocchi et al. 2017), the results achieved in our study reflected correlations between the latent factors that did not exceed 0.80 in any case and HTMT values below 0.80, these data support the discriminant validity of the instrument. Similarly, the results of the reliability analysis coincided with previous studies of the IBQ and IBQ‐Self (Camire et al. 2020; Rocchi et al. 2017), where values above 0.70 were obtained for all the variables that make up the instrument.

Finally, it is worth highlighting the results achieved in the relationships established in the SEM. In the SEM, it can be observed how the interpersonal behaviors of frustration (coaches and athletes) correlated positively with each other, as did the correlation with the interpersonal behaviors of satisfaction (coaches and athletes). This dataset provides advances in SDT (Ryan and Deci 2008), in that it highlighted a substantial nuance in the social context of the athletes in alienating their perception from those of the coach's self‐perception. Specifically, the alignment of the direction of the perceived psychological needs of support/frustration in either direction suggests a “needs‐interested” profile of athletes who may feel that their needs are either supported or frustrated by their coach, and that this perception is mutual (Bhavsar et al. 2019).

As for the correlations of the interpersonal behaviors of satisfaction with respect to those of frustration, these were negative. These results are supported by previous analyses conducted in the CFA. Furthermore, supportive interpersonal behaviors were positively related to satisfaction of basic psychological needs and negatively related to frustration of basic psychological needs. As for frustrating interpersonal behaviors, they were negatively related to the satisfaction of basic psychological needs and positively related to the frustration of basic psychological needs. These relationships are in line with the postulates of SDT and with the study conducted by Rocchi and Pelletier (2018), where they showed that when coaches and athletes agreed on supportive behaviors, need satisfaction was promoted, and in the case of frustration behaviors, need frustration was predicted. Similarly, previous studies showed that supportive coach behaviors, as perceived by athletes, positively predicted need satisfaction and negatively predicted need frustration (Trigueros et al. 2023).

Finally, SEM results showed that need satisfaction positively predicted motivation towards physical activity and sport, whereas need frustration negatively predicted it. In turn, motivation towards physical activity and sport positively predicted intention to be physically active. These results are in line with previous research in the field of SDT, where need satisfaction has been shown to play a contributory role by showing a significant relationship with motivation and motivation with intention to be physically active (Manninen et al. 2022). Therefore, it would be expected, both from its wording of meaning and from previous relationships studied, that within the proposed model the satisfaction of psychological needs would present similar behavior and would positively predict motivation, as in the study by Balaguer et al. (2020) where it is highlighted that the satisfaction of needs would lead to internal motivation, and therefore, to favor constructs related to the adoption of active physical activity habits. In contrast, need frustration has been shown to play a disincentive role in relation to motivation and intention to be physically active. Previous studies such as the one conducted by Behzadnia (2021) highlight that frustration of needs would lead to a decrease in internal motivation, and thus, to disfavor constructs related to the adoption of active physical activity habits. In the same way, the study by Morbée et al. (2020) highlighted that the control exercised by the coach increases the frustration of the athlete's needs, which reduces his or her motivation and involvement in sport and physical activity.

### 4.1 Limitations

Despite the contributions of this study, several limitations must be acknowledged to properly interpret the results.

First, the cross‐sectional design of the study prevents any strict causal inferences from being drawn. Although the SEM supports the hypothesized directional paths based on SDT, all data were collected at a single time point. Consequently, we cannot determine the temporal precedence of the variables. Future research should employ longitudinal or experimental designs to firmly establish the causality of the relationships between coaches’ interpersonal behaviors, athletes’ basic psychological needs, motivation, and future intentions to be physically active. Second, regarding the sample, although representative, it is limited to the outcome of the specific cultural context of Southern Spain (Andalusia). Therefore, caution should be exercised when generalizing these findings to other regions or countries with different sporting traditions. Third, a potential “social desirability bias” cannot be ruled out, particularly in the coaches’ self‐reported measures, as they may unintentionally overestimate their supportive behaviors compared to the athletes’ actual perceptions. Four, although the selected SEM showed excellent fit indices, it is recognized that equivalent models could exist that also explain the data (Hershberger and Marcoulides 2006). Fifth, the average experience of the coaches in our sample was relatively modest (*M* = 3.45 years). This is a limitation, as establishing a deeply stable and consolidated interpersonal coaching style may require more years of professional practice. Future studies should aim to include more experienced coaches to verify if the structural relationships hold. Finally, regarding the relationship between coaches’ and athletes’ perceptions, the present study evaluated global associations using SEM. Because the data were not structured as matched dyadic pairs (specific athletes nested within their specific coaches), true congruence analysis methods—such as the actor–partner interdependence model (APIM) or polynomial regression—could not be applied. Future research should employ dyadic designs and these specific methodologies to further examine strict coach–athlete congruence.

## 5 Conclusions

The present study provides support for its primary objective and consistent with the initial hypotheses. First, the psychometric analysis validates the adaptation of the questionnaire to the Spanish context, confirming that the inclusion of novelty as a fourth dimension is not only theoretically viable but statistically robust. This supports the premise that modern coaching analysis requires a broader spectrum beyond the traditional three basic psychological needs.

Second, the hypothesis regarding the interaction between perspectives is confirmed: The model demonstrates that there is a significant relationship between how coaches perceive their own behavior and how athletes perceive it. However, the study highlights that these views do not always perfectly align, suggesting that strong association is a key predictor for the satisfaction of psychological needs. Consequently, the results contribute significantly to SDT by showing that the coach's supportive interpersonal behavior—whether self‐perceived or observed—is a key associated factor. It is effectively in the coach's hands to foster well‐being climates (Howie et al. 2020), but this requires an awareness of the potential gap between their intention (self‐report) and the athletes’ reception. Future practical interventions should focus on narrowing this gap to ensure adaptive behaviors (Schlechter et al. 2017).

## Author Contributions

Conceptualization: **Rubén Trigueros** and **José M. Aguilar‐Parra**. Data curation: **Carmen Fernandez‐Ortega** and **Maria del Mar Miralles‐Dionis**. Formal analysis: Rubén Trigueros. Funding acquisition: Carmen Fernandez‐Ortega and Maria del Mar Miralles‐Dionis. Investigation: José M. Aguilar‐Parra. Methodology: José M. Aguilar‐Parra and Rubén Trigueros. Project administration: Rubén Trigueros. Resources: Carmen Fernandez‐Ortega, Maria del Mar Miralles‐Dionis, and Rubén Trigueros. Software: Rubén Trigueros. Writing – original draft: Carmen Fernandez‐Ortega and Rubén Trigueros. Writing – review and editing: José M. Aguilar‐Parra and Maria del Mar Miralles‐Dionis. All authors have read and agreed to the published version of the manuscript.

## Funding

The authors have nothing to report.

## Ethics Statement

The present study was approved by the Bioethics Committee of the University of Almeria (REF: UALBIO2024/007). In addition, all procedures performed in the studies with human participants were in accordance with the ethical standards of the institutional and/or national research committee and with the 1964 Helsinki declaration and its subsequent amendments or comparable ethical standards.

## Consent

All participants in the study agreed to participate freely and without any external pressure or reward. Informed consent was obtained from all individual participants included in the study.

## Conflicts of Interest

The authors declare no conflicts of interest.

## Supporting information




**Supplementary Materials**: brb371419‐sup‐0001‐SuppMat.docx

## Data Availability

The datasets generated during and/or analyzed during the current study are not publicly available due to the fact that we do not have the consent of the study participants but are available from the corresponding author on reasonable request.
