# Supplementary material for: Validation and Adaptation of the Interpersonal Behaviors Questionnaire in the Spanish Sport Context: The Inclusion of Novelty and Analysis of Coach–Athlete Perspectives
Source: Brain Behav. 2026 Apr 16;16(4):e71419. doi: 10.1002/brb3.71419 (PMC13087503; doi:10.1002/brb3.71419)
Supplement: Supplementary file 1 — Supplementary Materials: brb371419‐sup‐0001‐SuppMat.docx [file BRB3-16-e71419-s001.docx]

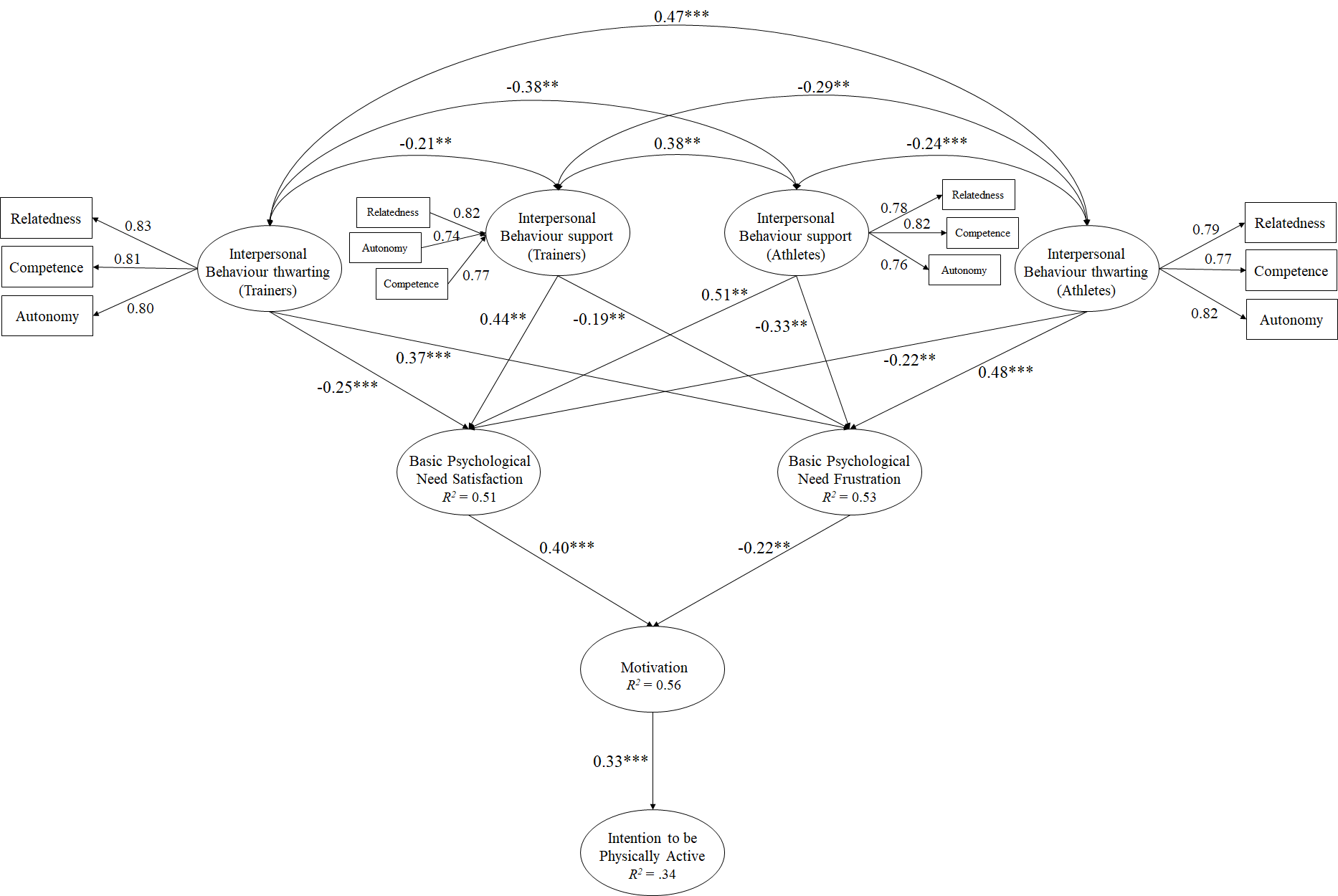


Figure S1: First alternative model.


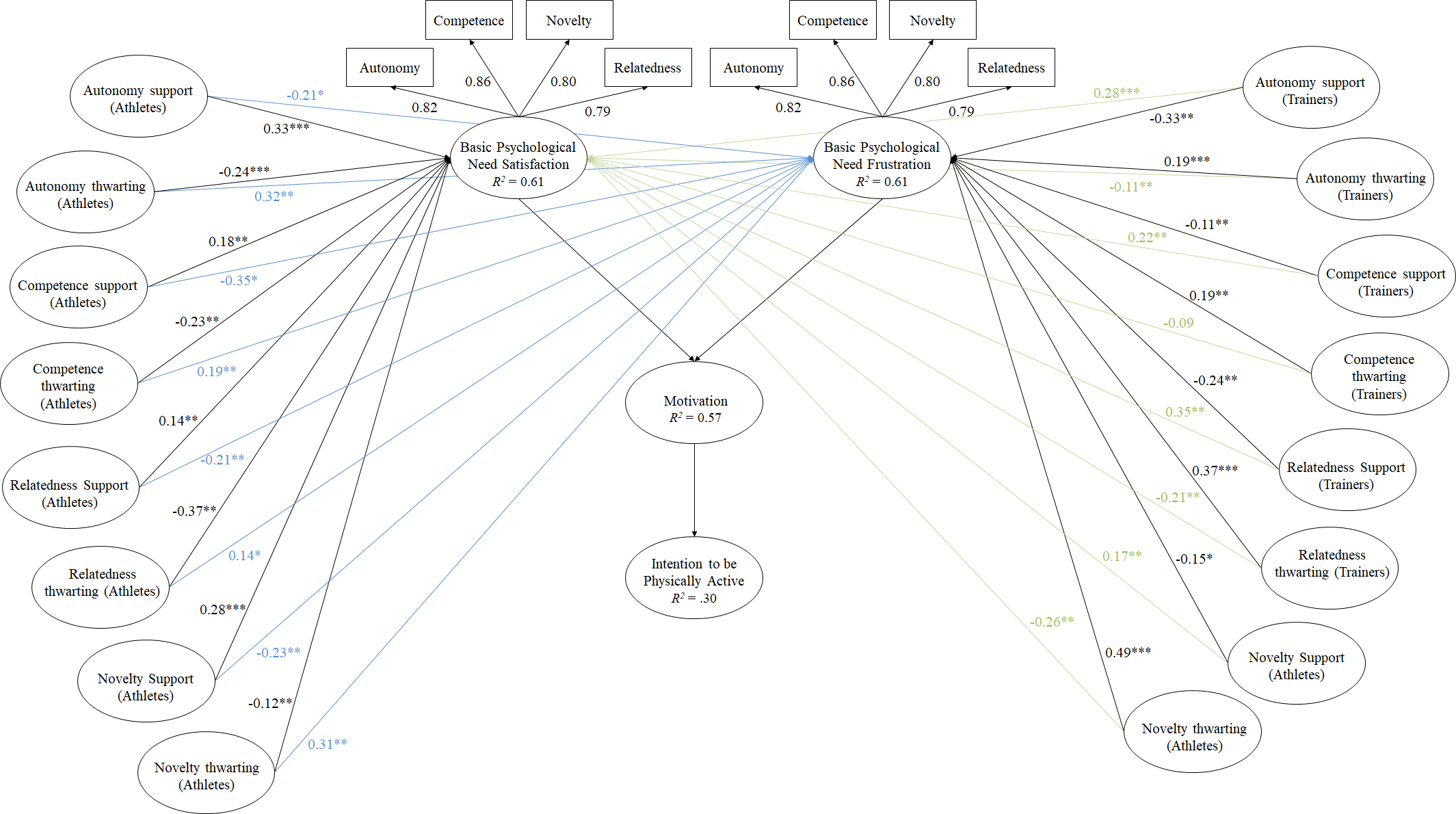


Figure S2: 2nd alternative model.

| Table S. Correlations between IBQ and IBQ-Self factors belonging to the 2nd alternative model. | | | | | | | | | | | | | | | | |
| --- | --- | --- | --- | --- | --- | --- | --- | --- | --- | --- | --- | --- | --- | --- | --- | --- |
| Factors | 1 | 2 | 3 | 4 | 5 | 6 | 7 | 8 | 9 | 10 | 11 | 12 | 13 | 14 | 15 | 16 |
| 1. Autonomy thwarting (Athletes) | - | .22*** | .31*** | .10* | -.44** | -.17** | -.27*** | -.08 | .55** | .20** | .16* | .24* | -.55** | -.04 | -.21* | -.47*** |
| 2. Novelty thwarting (Athletes) |  | - | .29** | .18** | -.30* | -.57*** | -.18** | -.10* | .11 | .78*** | .14* | .25** | -.10 | -.11* | .07 | -.19* |
| 3. Competence thwarting (Athletes) |  |  | - | .20** | -.28** | -.45** | -.61** | -.33** | .26** | .29* | .60*** | .11 | -.32** | -.23** | -.32* | -.24** |
| 4. Relatedness thwarting (Athletes) |  |  |  | - | -.13** | .07 | -.23** | -.45*** | .17* | .07* | .15** | .49** | .14** | -.35*** | -.25** | -.36** |
| 5. Autonomy support (Athletes) |  |  |  |  | - | .24** | .35** | .42* | -.54** | -.30* | .19** | .24* | .67*** | .29* | .35** | .45** |
| 6. Novelty support (Athletes) |  |  |  |  |  | - | .42*** | .19** | -.29* | -.61** | -.26** | -.14** | .16 | .71*** | .10* | .17* |
| 7. Competence support (Athletes) |  |  |  |  |  |  | - | .21** | -.11* | -.25* | -.47*** | -.37*** | -.34* | .13 | .02 | .15 |
| 8. Relatedness support (Athletes) |  |  |  |  |  |  |  | - | -.27*** | -.18** | -.21 | -.52*** | -.20*** | .26*** | .38** | .29*** |
| 9. Autonomy thwarting (Coaches) |  |  |  |  |  |  |  |  | - | .30*** | -.25* | .20** | -.43*** | -.22** | -.04 | -.50** |
| 10. Novelty thwarting (Coaches) |  |  |  |  |  |  |  |  |  | - | .16** | -.12 | -.37** | -.51** | -.11 | -.06 |
| 11. Competence thwarting (Coaches) |  |  |  |  |  |  |  |  |  |  | - | .27** | .14* | -.28* | -.44*** | -.18* |
| 12. Relatedness thwarting (Coaches) |  |  |  |  |  |  |  |  |  |  |  | - | .22*** | -.11* | -.22* | -.36*** |
| 13. Autonomy support (Coaches) |  |  |  |  |  |  |  |  |  |  |  |  | - | .19* | .25* | .15* |
| 14. Novelty support (Coaches) |  |  |  |  |  |  |  |  |  |  |  |  |  | - | .17** | .08 |
| 15. Competence support (Coaches) |  |  |  |  |  |  |  |  |  |  |  |  |  |  | - | .20* |
| 16. Relatedness support (Coaches) |  |  |  |  |  |  |  |  |  |  |  |  |  |  |  | - |
| Note: Correlations were calculated from the scores of the standardized regression weights; *p<* .001***; *p<* .01**; *p<* .05* | | | | | | | | | | | | | | | | |

Figure S2: Second alternative model.
